# Normative Values for Heart Rate Variability Parameters in School-Aged Children: Simple Approach Considering Differences in Average Heart Rate

**DOI:** 10.3389/fphys.2018.01495

**Published:** 2018-10-24

**Authors:** Jakub S. Gąsior, Jerzy Sacha, Mariusz Pawłowski, Jakub Zieliński, Piotr J. Jeleń, Agnieszka Tomik, Tomasz M. Książczyk, Bożena Werner, Marek J. Dąbrowski

**Affiliations:** ^1^Faculty of Health Sciences and Physical Education, Kazimierz Pulaski University of Technology and Humanities in Radom, Radom, Poland; ^2^Cardiology Clinic of Physiotherapy Division of the 2nd Faculty of Medicine, Medical University of Warsaw, Warsaw, Poland; ^3^Faculty of Physical Education and Physiotherapy, Opole University of Technology, Opole, Poland; ^4^Department of Cardiology, University Hospital, Faculty of Natural Sciences and Technology, University of Opole, Opole, Poland; ^5^Department of Biophysics and Human Physiology, Medical University of Warsaw, Warsaw, Poland; ^6^Interdisciplinary Centre for Mathematical and Computational Modelling, University of Warsaw, Warsaw, Poland; ^7^Department of Pediatric Cardiology and General Pediatrics, Medical University of Warsaw, Warsaw, Poland

**Keywords:** heart rate, heart rate variability, heart rate correction, children and adolescents, normative values, reference values, autonomic nervous system, autonomic cardiac control

## Abstract

**Background:** Heart rate variability (HRV) analysis is a clinical tool frequently used to characterize cardiac autonomic status. The aim of this study was to establish normative values for short-term HRV parameters by considering their main determinants in school-aged children.

**Methods:** Five-minute electrocardiograms were taken from 312 non-athlete children (153 boys) at age of 6 to 13 years for computation of conventional time- and frequency-domain HRV parameters. Heart rate (HR), respiratory rate, age, body mass index, and sex were considered as their potential determinants. Multiple regression analysis revealed that HR was the principal predictor of all standard HRV indices. To develop their universal normative limits, standard HRV parameters were corrected for prevailing HR.

**Results:** The HRV correction for HR yielded the parameters which became independent on both sex and HR, and only poorly dependent on age (with small effect size). Normal ranges were calculated for both time- and frequency-domain indices (the latter computed with either fast Fourier transform and autoregressive method). To facilitate recalculation of standard HRV parameters into corrected ones, a calculator was created and attached as a Supplementary Material that can be downloaded and used for both research and clinical purposes.

**Conclusion:** This study provides HRV normative values for school-aged children which have been developed independently of their major determinants. The calculator accessible in the Supplementary Material can considerably simplify determination if HRV parameters accommodate within normal limits.

## Introduction

Since the early 1970s, when power spectral analysis was applied to explore the physiological basis of intermittent variations in heart rate (HR) (Hyndman et al., 1971; Chess et al., 1975; Akselrod et al., 1981; Billman, 2011; Ernst, 2017b), a large number of studies addressing heart rate variability (HRV) have been published (Xhyheri et al., 2012). Reduced HRV corresponds to the autonomic nervous system (ANS) imbalance and may be associated with worse prognosis (particularly increased mortality) in various disease states among adults (Kleiger et al., 1987; Dekker et al., 2000; Stein et al., 2005; Thayer et al., 2010; Ernst, 2017a). Also in children, depressed HRV may be related to some cardiac (Massin and von Bernuth, 1998; Heragu and Scott, 1999; Bakari et al., 2013) and non-cardiac disorders (Chessa et al., 2002; Birch et al., 2012). However, to diagnose the ANS imbalance normative or reference values for HRV indices need to be established. Nevertheless, only a limited number of studies reported such normative/reference HRV values in pediatric populations (Goto et al., 1997; Massin and von Bernuth, 1997; Umetani et al., 1998; Silvetti et al., 2001; Rêkawek et al., 2003; Longin et al., 2009; Michels et al., 2013; Seppälä et al., 2014; Jarrin et al., 2015; Sharma et al., 2015; Bobkowski et al., 2017). Usually, authors presented their normal values for children by categorizing them according to age and/or sex (Massin and von Bernuth, 1997; Silvetti et al., 2001; Rêkawek et al., 2003; Sharma et al., 2015), however, HRV parameters are also significantly associated with other factors, like HR (Goto et al., 1997; Massin and von Bernuth, 1997; Jarrin et al., 2015), respiration (Grossman and Taylor, 2007; Beda et al., 2014; Quintana et al., 2016a,b; Shader et al., 2017; Shaffer and Ginsberg, 2017), physical activity (Oliveira et al., 2017), or weight status (Eyre et al., 2014). Since HRV is primarily HR dependent (Fleiss et al., 1992; Goto et al., 1997; Massin and von Bernuth, 1997; Sacha and Grzeszczak, 2001; Sacha and Pluta, 2005a,b; Sacha and Pluta, 2008; Burr et al., 2006; Nieminen et al., 2007; Billman, 2013; Sacha, 2013, 2014a,b,c; Sacha et al., 2013a,b,c; Monfredi et al., 2014; Billman et al., 2015; Gąsior et al., 2015; Jarrin et al., 2015), any alteration in average HR automatically changes HRV. Consequently, a comparison of HRV corresponding to different HR may be biased (Sacha and Grzeszczak, 2001; Nieminen et al., 2007; Sacha and Pluta, 2008). Mathematical methods removing the HRV dependence on HR (i.e., the HRV correction for prevailing HR) (Hayano et al., 1990, 1991; Sacha and Grzeszczak, 2001; Sacha and Pluta, 2008; Sacha et al., 2013b,c; Monfredi et al., 2014; Estévez-Báez et al., 2015; van Roon et al., 2016) should allow to draw objective conclusions when comparing HRV associated with various HR (Sacha, 2013, 2014a,b,c; Monfredi et al., 2014; Billman et al., 2015). So far, only one study dealing with normal ranges of HRV employed the HRV correction for HR, however, authors presented normal values only for two time domain HRV markers derived from 10-s ECGs (van den Berg et al., 2018). Recently, researchers applying such a correction have demonstrated a significant interaction between HRV and HR in healthy pediatric populations (Gąsior et al., 2015; Bjelakovic et al., 2017; Herzig et al., 2017). It has been shown that while standard HRV may remain constant in different age subgroups, corrected HRV decreases with age along with a decrease in average HR.

Another important factor, usually not accounted for in HRV normative studies but significantly influencing both HR and HRV, is breathing (Quintana et al., 2016a; Shader et al., 2017). The respiratory rate declines from birth to early adolescence (Fleming et al., 2011), and consequently such changes may influence HRV (Billman, 2011; Quintana and Heathers, 2014; Quintana et al., 2016a,b; Laborde et al., 2017).

The aims of the present study were to determine the main determinants of HRV in healthy school-aged children, and by incorporating the significant determinants to define normative values for both time- and frequency-domain HRV indices.

## Materials and Methods

### Study Population

The whole study group consisted of 346 children of both sexes. The inclusion criteria to this study were as follows: (i) age between 6 and 13 years, (ii) absence of diseases and/or regular use of medications affecting the cardiopulmonary system and/or interfering with the autonomic nervous system, and (iii) not being an active athlete in any sports. The parents/legal guardians were interviewed about children’s diseases and/or medications (the school health records concerning health status were additionally verified). Fifteen subjects were excluded from the analysis due to suspicion of cardiac/non-cardiac diseases. The group participated in an earlier study, its details and description of the procedures imposed before or during the proper electrocardiographic examinations have been published elsewhere (Gąsior et al., 2015). Since there are data indicating an association of cardiac autonomic function with physical activity in children and adolescents (Oliveira et al., 2017), we excluded 19 active athletes (Araújo and Scharhag, 2016) from the present study providing normative values. Consequently, 312 healthy Caucasian children of both sexes between the ages of 6 and 13 years were enrolled in the final analysis. The group was divided into four subgroups according to age: (I) 6 ≤ age < 8 years; (II) 8 ≤ age < 10 years; (III) 10 ≤ age < 12 years; and (IV) 12 ≤ age < 14 years. This classification is in accordance with other studies where participants younger than 10 years were considered as children and those between 10 and 14 years as preadolescents or early adolescents (Frigerio et al., 2006; Cysarz et al., 2011; Varlinskaya et al., 2013). The study was approved by the University Bioethical Committee and followed the rules and principles of the Helsinki Declaration, as well as all parents or legal guardians gave their informed written consent.

### Body Mass Status Measurement

The body mass status was measured using Body Mass Index (BMI) defined as body mass in kilograms divided by height in meters squared. BMI is widely used for assessing the weight status of pediatric participants since it can identify those who are overweight, at risk of being overweight, or underweight based on age and sex (Barlow and Dietz, 1998).

### Electrocardiographic (ECG) Recordings and HRV Analysis

Twelve-lead, 6-min ECG recordings (sampling frequency = 1000 Hz) were performed using a portable PC with integrated software system (Custo cardio 100 12-channel PC ECG system; Custo med GmbH, Ottobrunn, Germany) during regular school days between 8 a.m. and 2 p.m. in a supine position. For heart rate stabilization, children were placed in a supine position for 5 min before the examination. All ECG recordings were preprocessed before HRV analysis, i.e., they were visually inspected for potential non-sinus or aberrant beats and such erroneous beats were properly corrected (interpolation of degree zero) (Peltola, 2012). Time- and frequency-domain HRV analyses were performed from 5-min ECG recordings by using Kubios HRV Standard 3.0.2 software (University of Eastern Finland, Kuopio, Finland) (Tarvainen et al., 2014, 2017). Standard deviation of R-R intervals (SDNN), root mean square of successive R-R interval differences (RMSSD), and pNN50, which denotes percent of R-R intervals differing >50 ms from the preceding one, were determined. Before calculating spectral HRV parameters, smoothness priors based detrending approach was applied (smoothing parameter, Lambda value = 500) (Tarvainen et al., 2002), and then R-R interval series were transformed to evenly sampled time series with 4-Hz resampling rate. The detrended and interpolated R-R interval series were used to compute HRV spectra by employing fast-Fourier-transform (FFT) with Welch’s periodogram method (300 s window width without overlap) and by using the autoregressive model (AR) with model order set at 16 (Boardman et al., 2002). The following spectral components were distinguished: very low frequency (VLF, 0–0.04 Hz), low frequency (LF, 0.04–0.15 Hz), high frequency (HF, 0.15–0.50 Hz), and total power [(in two versions, i.e., TP_1_, 0–0.5 Hz (VLF+LF+HF) and TP_2_, 0.04–0.50 Hz (LF+HF)] in absolute units (ms^2^), as well as nLF, nHF in normalized units (nu) (Task Force, 1996).

### ECG Derived Respiration

The respiratory rate (RespRate) was calculated from ECG recordings according to Sinnecker et al. (2014). The time series of the QRS amplitude for all 12 ECG leads were calculated and then local maxima (i.e., data points with values greater than both the preceding and the following data point) were established. Then, the mean interval between consecutive local maxima for each time series was computed and the reciprocal value of the mean maximum-to-maximum interval was obtained. The median of these reciprocal values over all-time series represented RespRate used in this analysis (Sinnecker et al., 2014).

### HRV Correction

Since HRV is dependent on HR due to both physiological (i.e., the autonomic nervous system influence) and mathematical (i.e., the inverse non-linear relationships between HR and R-R interval) reasons, it is hard to ascertain if clinical significance of HRV comes from the variability or from HR (Sacha and Pluta, 2008) – it is even more important in populations where average HR is changing, like in children during their grow and development. Therefore, to investigate the “objective” variability, one should get rid of the HR impact on HRV. To do so, one should correct the HRV for the prevailing HR. The correction procedure employed in this study relied on the division or multiplication of standard HRV indices by different powers of their corresponding mean R-R interval (mRR) – the higher HRV dependence on HR, the higher power of mRR must be used to get rid of this dependence. Specifically, if HRV parameters revealed negative relation with HR (i.e., SDNN, RMSSD, pNN50, VLF, LF, HF, TP_1_, TP_2_, and nHF), they should be divided by the suitable power of mRR in order to become HR independent, however, if the HRV parameters are positively related with HR (i.e., nLF and LF/HF), they must be multiplied by the appropriate power of mRR – this way we were able to remove both physiological and mathematical HRV dependence on HR (Sacha et al., 2013b,c).

### Statistical Analysis

The Kolmogorov–Smirnov test was used to assess the normality of the data distribution. Normally distributed values were presented as mean ± standard deviation, but others as median and interquartile range (IQR). Accordingly, Pearson’s correlation coefficient (r) or Spearman’s rank correlation coefficient (R) were used to assess the relationship between variables, and Student’s *t*-test or non-parametric Mann–Whitney test were employed to determine differences. The Pearson’s chi-squared test was applied to determine differences between variables prevalence in multiple groups. Values of the standard and corrected HRV parameters were compared between four age subgroups by using Kruskal–Wallis (K-W) test and analysis of variance (ANOVA), respectively. Multiple regression analysis was carried out to identify independent determinants of standard and corrected HRV parameters – variables with a skewed distribution were logarithmically transformed (ln). To avoid multicollinearity, redundant variables were removed from the multivariate regression models in the case of pairwise correlations between continuous variables (Slinker and Glantz, 2008). The effect size was measured by calculating Cohen’s *f*^2^ within a multiple regression model (Selya et al., 2012). The *f*^2^ were calculated for a combined prediction of the model, and Cohen’s *f*^2^ variation was computed to measure the local effect size (Cohen, 1992; Selya et al., 2012). According to Cohen’s guidelines, *f*^2^ ≥ 0.02, *f*^2^ ≥ 0.15, and *f*^2^ ≥ 0.35 represent small, medium, and large effect sizes, respectively (Cohen et al., 2003). The threshold probability of *p* < 0.05 was taken as the level of statistical significance. Since all HRV parameters were not normally distributed, normative ranges have been given as medians with the 5th and 95th percentiles. Statistical calculations were performed by using the software STATISTICA 10 (StatSoft, Inc., Software, Tulsa, OK, United States). Graphs were created with GraphPad PRISM^®^ Version 5.0 (GraphPad Software, Inc., San Diego, CA, United States) and Microsoft Office Excel 2007 (Microsoft Corporation, Silicon Valley, CA, United States).

## Results

The group of 312 healthy non-athlete children (153 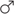, 159 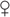) at age of 6 to 13 years (median: 10.1 years, IQR: 8.4–11.8 years) were analyzed in the present study. The group’s characteristics were: median body mass, 34.1 kg (IQR: 27.7–45.1 kg); median height, 140.0 cm (IQR: 129.5–152.5 cm); median BMI, 17.3 kg/m^2^ (IQR: 15.7–20.1 kg/m^2^); mean HR, 84.4 ± 10.6 bpm (range: 54.7–113.8 bpm); and mean respiratory rate (RespRate), 22.7 ± 3.2 bpm (range: 12.4–30.7 bpm).

Comparing with boys, girls presented significantly higher HR, lower values of all time-domain HRV parameters, i.e., SDNN, RMSSD, pNN50, and the following frequency-domain parameters (calculated with both FFT and AR): VLF, LF, TP_1_ (VLF+LF+HF), TP_2_ (LF+HF) (*p* < 0.05 for all). Boys and girls did not differ in RespRate (*p* = 0.64), BMI (*p* = 0.71), and the following spectral parameters: HF, LF/HF, nLF, and nHF (*p* > 0.05 for all).

**FIGURE 1:**
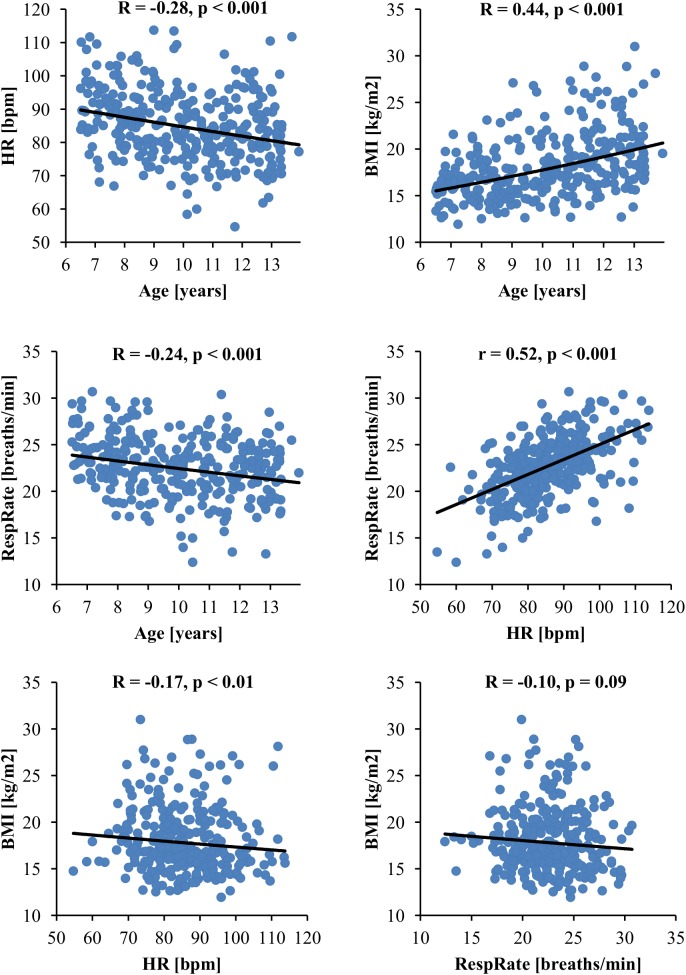
Association between variables selected as potential independent predictors of standard HRV parameters. BMI, body mass index; HR, heart rate; HRV, heart rate variability; RespRate, respiratory rate; R, Spearman’s rank correlation coefficient; r, Pearson’s correlation coefficient.

Heart rate, RespRate, age, BMI and sex were initially chosen as potential determinants of standard HRV parameters (Table 1 presents their correlations with HRV). All these variables were associated with each other with one exception, i.e., there was no significant correlation between RespRate and BMI (Figure 1). Since the association between RespRate and HR was stronger than between RespRate and HRV, as well as, the association between BMI and age was stronger than between BMI and HRV, (Figure 1 and Table 1), both RespRate and BMI were considered to be redundant for the multivariate analysis (collinearity tests confirmed this redundancy). Accordingly to the statistical assumptions of the multiple regression analysis (Kraha et al., 2012; Ray-Mukherjee et al., 2014) and taking into account the practicality of normative data in a clinical context, the following variables were included in the regression analysis: HR, age, and sex. Table 2 exhibits results of this analysis for time- and frequency-domain HRV parameters calculated with FFT – the results for HRV spectra estimated with AR were similar and are presented in Supplementary Table S1. The models accounted for 9–60% of the entire HRV variance, but importantly, HR was the strongest predictor for all analyzed standard HRV parameters. HR was the most powerful determinant in the whole group (Table 2 and Supplementary Table S1) and also within the age subgroups: 6–7, 8–9, 10–11, and 12–13 years (Supplementary Tables S2–S9).

**Table 1:** Correlations of standard time- and frequency-domain HRV parameters with heart rate, respiratory rate, age, and body mass index.

Standard HRV parameter	HR [bpm]		RespRate [breaths/min]		Age [years]		BMI [kg/m^2^]	
				
	*R*	*p*	*R*	*p*	*R*	*p*	*R*	*p*
SDNN [ms]	-0.64		-0.41		-0.03	0.61	-0.04	0.52
RMSSD [ms]	-0.72		-0.37		<-0.01	0.99	-0.04	0.45
pNN50 [%]	-0.75		-0.36		0.02	0.72	-0.04	0.54
_FFT_ VLF [ms^2^]	-0.42		-0.22		-0.07	0.23	0.04	0.50
_FFT_ LF [ms^2^]	-0.53		-0.31		-0.04	0.49	<0.01	0.97
_FFT_ HF [ms^2^]	-0.60		-0.44		-0.06	0.29	-0.07	0.22
_FFT_ TP_1_ (VLF+LF+HF) [ms^2^]	-0.60		-0.41		-0.06	0.28	-0.04	0.46
_FFT_ TP_2_ (LF+HF) [ms^2^]	-0.60		-0.41		-0.06	0.31	-0.05	0.42
_FFT_ LF/HF	0.25		0.26		0.05	0.36	0.10	0.08
_FFT_ nLF [nu]	0.25	<0.001	0.26	<0.001	0.05	0.36	0.10	0.08
_FFT_ nHF [nu]	-0.25		-0.26		-0.05	0.36	-0.10	0.08
_AR_ VLF [ms^2^]	-0.56		-0.33		-0.08	0.19	-0.02	0.68
_AR_ LF [ms^2^]	-0.56		-0.34		-0.02	0.77	0.01	0.81
_AR_ HF [ms^2^]	-0.63		-0.45		-0.03	0.57	-0.06	0.28
_AR_ TP^1^ (VLF+LF+HF) [ms^2^]	-0.63		-0.43		-0.03	0.64	-0.03	0.57
_AR_ TP^2^ (LF+HF) [ms^2^]	-0.63		-0.43		-0.02	0.67	-0.03	0.55
_AR_ LF/HF	0.26		0.25		0.05	0.36	0.10	0.06
_AR_ nLF [nu]	0.26		0.25		0.05	0.36	0.10	0.06
_AR_ nHF [nu]	-0.26		-0.25		-0.05	0.37	-0.10	0.06


*FFT denotes that a given HRV parameter was calculated by using Fast Fourier Transform, while AR indicates that the HRV spectrum was calculated with autoregressive method. R, Spearman’s rank correlation coefficient.*

**Table 2:** Determinants of standard time- and frequency-domain (calculated with FFT) HRV parameters.

Standard HRV parameter	Determinant	Parameters of multiple regression analysis					
		
		β	*p*	Partial correlation	Multiple *R*^2^	*F*-test	*p*
SDNN (ln)	HR	-0.73	<0.001	-0.70	0.50	102.3	<0.001
	Age (ln)	-0.23	<0.001	-0.30			
	Sex	0.03	0.54	0.04			
RMSSD (ln)	HR	-0.81	<0.001	-0.77	0.60	153.8	<0.001
	Age (ln)	-0.24	<0.001	-0.35			
	Sex	0.01	0.74	0.02			
pNN50 (ln)	HR	-0.77	<0.001	-0.72	0.53	113.0	<0.001
	Age (ln)	-0.23	<0.001	-0.30			
	Sex	-0.02	0.59	-0.03			
VLF (ln)	HR	-0.49	<0.001	-0.47	0.24	31.9	<0.001
	Age (ln)	-0.19	<0.001	-0.21			
	Sex	0.05	0.30	0.06			
LF (ln)	HR	-0.61	<0.001	-0.58	0.36	57.3	<0.001
	Age (ln)	-0.20	<0.001	-0.23			
	Sex	0.08	0.09	0.10			
HF (ln)	HR	-0.70	<0.001	-0.67	0.46	85.9	<0.001
	Age (ln)	-0.26	<0.001	-0.32			
	Sex	<0.01	0.88	<0.01			
TP_1_ (VLF+LF+HF) (ln)	HR	-0.69	<0.001	-0.67	0.45	85.4	<0.001
	Age (ln)	-0.24	<0.001	-0.30			
	Sex	0.03	0.43	0.05			
TP_2_ (LF+HF) (ln)	HR	-0.70	<0.001	-0.67	0.46	86.1	<0.001
	Age (ln)	-0.24	<0.001	-0.30			
	Sex	0.03	0.46	0.04			
LF/HF (ln)	HR	0.31	<0.001	0.29	0.09	10.4	<0.001
	Age (ln)	0.15	<0.01	0.15			
	Sex	0.10	0.08	0.10			
nLF	HR	0.31	<0.001	0.30	0.10	10.8	<0.001
	Age (ln)	0.16	<0.01	0.15			
	Sex	0.10	0.07	0.10			
nHF	HR	-0.31	<0.001	-0.30	0.10	10.8	<0.001
	Age (ln)	-0.15	<0.01	-0.15			
	Sex	-0.10	0.07	-0.10			


Since HR was the strongest predictor for all standard HRV parameters, in order to calculate the normative values, the overall study group was classified into four quartile subgroups based on HR value: 1st quartile (Q1: <25%), 54.7–77.8 bpm; 2nd quartile (Q2: 25–50%), >77.8–84.3 bpm; 3rd quartile (Q3: 50–75%), >84.3–92.0 bpm; and 4th quartile (Q4: >75%), >92.0–113.8 bpm. The number of participants equaled to 78 in each subgroup: Q1 (male/female), 45/33; Q2, 41/37; Q3, 32/46; and Q4, 35/43. There was no significant difference in sex distribution in these subgroups (Pearson’s Chi-squared = 5.27, *p* = 0.15). There were statistically significant differences in all standard HRV parameters between the HR quartile subgroups (results of K-W test ranged between 26.6 and 160.8 with *p* < 0.001 for all). Normative values of standard time- and frequency-domain HRV parameters according to HR quartiles are presented in Table 3.

**Table 3:** Normative standard time- and frequency-domain HRV parameters values according to heart rate quartiles are presented as median, 5th–95th percentiles.

Standard HRV parameter	Q1	Q2	Q3	Q4
SDNN [ms]	75 (38–118)	56 (29–95)	43 (25–80)	35 (16–54)
RMSSD [ms]	86 (42–137)	65 (31–107)	44 (22–89)	33 (15–55)
pNN50 [%]	54 (22–70)	42 (11–63)	23 (2–54)	10 (0–30)
_FFT_ VLF [ms^2^]	102 (30–389)	66 (19–220)	47 (13–138)	41 (8–216)
_FFT_ LF [ms^2^]	1479 (328–5162)	705 (162–3286)	546 (236–2562)	421 (75–1193)
_FFT_ HF [ms^2^]	3269 (681–9485)	1808 (396–5679)	912 (265–4647)	527 (96–1956)
_FFT_ TP_1_ (VLF+LF+HF) [ms^2^]	5096 (1178–13023)	2750 (777–8791)	1544 (563–6671)	1079 (222–2959)
_FFT_ TP_2_ (LF+HF) [ms^2^]	4994 (1129–12787)	2703 (760—-8608)	1493 (528–6558)	1043 (206–2924)
_FFT_ LF/HF	0.48 (0.17–1.31)	0.54 (0.12–1.42)	0.67 (0.24–1.97)	0.87 (0.21–2.45)
_FFT_ nLF [nu]	32 (15–57)	35 (11–59)	40 (20–66)	46 (18–71)
_FFT_ nHF [nu]	68 (43–86)	65 (41–89)	60 (34–81)	54 (29–82)
_AR_ VLF [ms^2^]	205 (68–516)	122 (32–397)	88 (34–284)	61 (18–191)
_AR_ LF [ms^2^]	1411 (376–4547)	828 (146–2702)	559 (195–2029)	387 (92–1216)
_AR_ HF [ms^2^]	3063 (717–5454)	1939 (396–6127)	988 (261–4325)	576 (85–1706)
_AR_ TP_1_ (VLF+LF+HF) [ms^2^]	5061 (1444–12919)	2936 (791–8646)	1778 (580–6636)	1118 (202–2774)
_AR_ TP_2_ (LF+HF) [ms^2^]	4826 (1248–12277)	2800 (734–8404)	1662 (542–6502)	1015 (184–2638)
_AR_ LF/HF	0.44 (0.17–1.32)	0.55 (0.15–1.19)	0.61 (0.26–1.83)	0.73 (0.23–2.29)
_AR_ nLF [nu]	30 (14–57)	35 (13–54)	38 (20–65)	42 (19–70)
_AR_ nHF [nu]	69 (43–86)	65 (46–87)	62 (35–79)	58 (30–81)


*FFT denotes that a given HRV parameter was calculated by using Fast Fourier Transform, while AR indicates that the HRV spectrum was calculated with autoregressive method. HR quartiles for overall study group: (Q1), 54.7–77.8 bpm; (Q2), >77.8–84.3 bpm; (Q3), >84.3–92.0 bpm; (Q4), >92.0–113.8 bpm.*

However, there was a significant overlap in the normative ranges between consecutive HR quartile subgroups which makes such data impractical. Therefore, considering the fact that HR was the most influential factor determining all standard HRV parameters, we excluded its impact by correcting the standard HRV parameters for their prevailing HR. This way we were able to get normative values for so called corrected HRV, i.e., the variability independent on average HR. Indeed, standard time-domain HRV parameters lost their dependence on HR after dividing SDNN, RMSSD, and pNN50 by mRR to the power: 2.2, 3.0, and 5.0, respectively. In spectral analysis, VLF obtained by FFT and AR lost its dependence on HR after dividing by mRR to the power 3.0 and 4.0, respectively. Other frequency-domain HRV indices lost their association with HR after dividing LF, HF, TP_1_ (VLF+LF+HF), TP_2_ (LF+HF), and nHF by mRR to the power: 4.0, 5.0, 5.0, 5.0, and 0.5, respectively (for both FFT and AR). The nLF and LF/HF (calculated with either FFT and AR) stopped being dependent on HR after multiplying by mRR to the power 1.0 (Sacha et al., 2013b). Correlation coefficients between HR and all corrected HRV parameters were not statistically significant and ranged between -0.09 and 0.08 (*p* > 0.10 for all).

In the multiple regression analysis, age turned out to be the strongest determinant of the corrected time- and frequency-domain HRV parameters calculated with FFT (Table 4) – the results were the same when HRV spectra were estimated with AR (the AR data are presented in Supplementary Table S10). However, the calculated models accounted for only 2–9% of the whole variance of the corrected HRV indices. The age contribution to this model was quite low (i.e., β-value ranged between -0.13 and -0.29), and the effect size for the combined model, as well as for the variables taken individually (age and sex) was small (*f*^2^ ≤ 0.091 for all). Therefore, practically there was no reason to establish normative values according to age, and we calculated normal limits for the overall study group (Table 5). Of note, due to division by high powers of mRR, the numbers in Table 5 are long (i.e., they present many zeros after the decimal place), consequently calculations and interpretations of such results may be uninterpretable in clinical situations, hence in the Supplementary Material one may find the Excel sheet calculator which recalculates standard HRV parameters into corrected ones and determines whether a given value of corrected HRV parameter is within normal limits (Supplementary Table S11, Sheet “Corrected HRV Calculator”).

**Table 4:** Determinants and Cohen’s *f*^2^ indexes for corrected time- and frequency-domain (calculated with FFT) HRV parameters.

Corrected HRV parameter	Det.	Parameters of multiple regression analysis						Cohen’s *f*^2^	
			
		β	*p*	PC	M. R2	*F*-test	*p*	Loc.	Comb.
corr-SDNN	Age (ln)	-0.24	<0.001	-0.24	0.06	10.6	<0.001	0.063	0.063
	Sex	0.07	0.21	0.07				0.005	
corr-RMSSD	Age (ln)	-0.28	<0.001	-0.28	0.08	13.9	<0.001	0.086	0.086
	Sex	0.06	0.27	0.06				0.004	
corr-pNN50	Age (ln)	-0.28	<0.001	-0.28	0.08	12.8	<0.001	0.083	0.083
	Sex	0.01	0.80	0.01				0.001	
corr-VLF	Age (ln)	-0.20	<0.001	-0.20	0.04	6.3	<0.01	0.041	0.039
	Sex	0.03	0.62	0.03				0.001	
corr-LF	Age (ln)	-0.20	<0.001	-0.21	0.06	9.1	<0.001	0.044	0.044
	Sex	0.12	<0.05	0.12				0.014	
corr-HF	Age (ln)	-0.20	<0.001	-0.20	0.04	6.7	<0.01	0.044	0.044
	Sex	0.01	0.95	0.01				0.001	
corr-TP_1_ (VLF+LF+HF)	Age (ln)	-0.25	<0.001	-0.25	0.06	10.2	<0.001	0.065	0.065
	Sex	0.03	0.54	0.04				0.001	
corr-TP_2_ (LF+HF)	Age (ln)	-0.24	<0.001	-0.24	0.06	9.8	<0.001	0.063	0.063
	Sex	0.03	0.54	0.04				0.001	
corr-LF/HF	Age (ln)	0.13	<0.05	0.13	0.02	3.3	<0.05	0.017	0.017
	Sex	0.06	0.26	0.06				0.004	
corr-nLF	Age (ln)	0.15	<0.01	0.15	0.03	5.4	<0.01	0.024	0.024
	Sex	0.11	0.06	0.11				0.011	
corr-nHF	Age (ln)	-0.15	<0.01	-0.15	0.03	5.0	<0.01	0.024	0.024
	Sex	-0.10	0.08	-0.11				0.008	


*Abbreviations: Det., determinant; PC, partial correlation; M. R2, multiple R2; Loc., local; Comb., combined. Corrected HRV parameters were calculated as follows: corr-SDNN, SDNN/mRRˆ2.2; corr-RMSSD, RMSSD/mRRˆ3.0; corr-pNN50, pNN50/mRRˆ5.0; FFT corr-VLF, VLF/mRRˆ3.0; AR corr-VLF, VLF/mRRˆ4.0; for both FFT and AR: corr-LF, LF/mRRˆ4.0; corr-HF, HF/mRRˆ5.0; corr-TP_1_, TP_1_/mRRˆ5.0; corr-TP_2_, TP_2_/mRRˆ5.0; corr-LF/HF, LF/HF^∗^mRRˆ1.0; corr-nLF, nLF^∗^mRRˆ1.0; and corr-nHF, nHF/mRRˆ0.5.*

**Table 5:** Normative values for corrected HRV parameters for the whole study group.

Corrected HRV parameters	Normative values		
	
	Median	5th percentile	95th percentile
corr-SDNN [ms^-1.2^]	2.6E-05	1.5E-05	4.6E-05
corr-RMSSD [ms^-2^]	1.4E-07	7.6E-08	2.6E-07
corr-pNN50 [%/ms^5^]	1.4E-13	2.0E-14	3.0E-13
_FFT_ corr-VLF [ms^-1^]	1.7E-07	4.4E-08	5.5E-07
_FFT_ corr-LF [ms^-2^]	2.9E-09	7.5E-10	9.8E-09
_FFT_ corr-HF [ms^-3^]	6.8E-12	1.7E-12	2.5E-11
_FFT_ corr-TP_1_ (VLF+LF+HF) [ms^-3^]	1.2E-11	3.6E-12	3.8E-11
_FFT_ corr-TP_2_ (LF+HF) [ms^-3^]	1.1E-11	3.3E-12	3.7E-11
_FFT_ corr-LF/HF [ms]	4.5E+02	1.5E+02	1.2E+03
_FFT_ corr-nLF [nu^∗^ms]	2.7E+04	1.3E+04	4.5E+04
_FFT_ corr-nHF [nu/ms^0.5^]	2.3E+00	1.4E+00	3.1E+00
_AR_ corr-VLF [ms^-2^]	4.3E-10	1.4E-10	1.2E-09
_AR_ corr-LF [ms^-2^]	2.7E-09	7.9E-10	8.8E-09
_AR_ corr-HF [ms^-3^]	7.3E-12	1.6E-12	2.7E-11
_AR_ corr-TP_1_ (VLF+LF+HF) [ms^-3^]	1.2E-11	3.7E-12	4.0E-11
_AR_ corr-TP_2_ (LF+HF) [ms^-3^]	1.1E-11	3.4E-12	3.8E-11
_AR_ corr-LF/HF [ms]	4.1E+02	1.4E+02	1.1E+03
_AR_ corr-nLF [nu^∗^ms]	2.6E+04	1.2E+04	4.3E+04
_AR_ corr-nHF [nu/ms^0.5^]	2.4E+00	1.5E+00	3.1E+00


*FFT denotes that a given HRV parameter was calculated by using Fast Fourier Transform, while AR indicates that the HRV spectrum was calculated with autoregressive method. Corrected HRV parameters were calculated as follows: corr-SDNN, SDNN/mRRˆ2.2; corr-RMSSD, RMSSD/mRRˆ3.0; corr-pNN50, pNN50/mRRˆ5.0; FFT corr-VLF, VLF/mRRˆ3.0; AR corr-VLF, VLF/mRRˆ4.0; for both FFT and AR: corr-LF, LF/mRRˆ4.0; corr-HF, HF/mRRˆ5.0; corr-TP_1_, TP_1_/mRRˆ5.0; corr-TP_2_, TP_2_/mRRˆ5.0; corr-LF/HF, LF/HF^∗^mRRˆ1.0; corr-nLF, nLF^∗^mRRˆ1.0; and corr-nHF, nHF/mRRˆ0.5.*

## Discussion

The aim of the present study was to define, by incorporating the significant determinants, normative values for HRV indices in healthy children. HRV is changing during childhood and adolescence which may reflect developmental alterations in the ANS activity. Thus, in order to follow these changes, normal values for HRV parameters need to be established. This study provides such normative quantities for short-term (i.e., 5 min) time- and frequency-domain HRV parameters based on a group of 312 school-aged children, i.e., at age of 6–13 years.

Since HRV is under influence of a number of factors, we conducted the multiple regression analysis which revealed that HR is the strongest predictor for all standard HRV parameters in the overall study group and in each analyzed age subgroups (Table 2 and Supplementary Tables S1–S9). Age was the second independent predictor but accounted only for a small percentage of the entire HRV variance. Interestingly, in the multivariate analysis sex did not independently determine HRV in our study population (Table 2 and Supplementary Table S1). The reason for this possibly comes from the observation that boys usually present significantly lower HR than girls and therefore sex differences in HRV may result from differences in HR. When considering HR, we could ignore differences between the sexes and establish normative HRV values only for subgroups categorized according to the quartiles of HR (Table 3). However, among such specified subgroups, confidence intervals significantly overlapped for most of HRV parameters, which may contest practicality of this approach. Hence, we calculated HRV independent on HR by correcting standard HRV indices for prevailing HR. In this method, we obtained so-called corrected HRV parameters, which reflect “objective” variability regardless whatever the average HR is. In the multiple regression analysis, age was significant predictor for such corrected HRV indices (Table 4 and Supplementary Table S10), nevertheless, its contribution to the whole variance of HRV was small, i.e., the models only accounted for up to 9% of the HRV variance and the age role in these models was modest (β values ranged between -0.29 and 0.13) as well as its effect size turned out to be small (*f*^2^ ≤ 0.091) (Table 4 and Supplementary Table S10). Therefore, we omitted age impact in these 6 to 13-year-old children and established normal limits for the entire study group (Table 5). The calculation of corrected HRV parameters and their assessment become simpler by using the file provided in the Supplementary Material, which recalculates standard HRV indices into corrected ones and automatically determines whether they accommodate within normal limits (Supplementary Table S11, Sheet “Corrected HRV Calculator”).

The influence of HR on HRV in children has also been recognized in other studies (Goto et al., 1997; Massin and von Bernuth, 1997; Jarrin et al., 2015; Bjelakovic et al., 2017; Herzig et al., 2017). Indeed, Jarrin et al. (2015), accounted the observation that HR is the strongest factor determining HRV and presented their normative values adjusted for HR, however, only in participants with a very narrow age range, i.e., 9–11 years (mean ± SD: 10.2 ± 0.3 years) (Jarrin et al., 2015). Yet, the problem of the strong association between HR and HRV can simply be resolved by correcting HRV parameters for prevailing HR (Sacha and Grzeszczak, 2001; Sacha and Pluta, 2005a,b, 2008; Sacha, 2013, 2014a,b,c; Sacha et al., 2013a,b,c, 2014; Gąsior et al., 2016) and this method turns out to be feasible and convenient also in children populations (Gąsior et al., 2015).

More and more clinical and methodological papers highlight that respiration should be taken into account, or at least monitored, in HRV studies (Brown et al., 1993; Williams and Lopes, 2002; Eckberg, 2003; Song and Lehrer, 2003; Yildiz and Ider, 2006; Beda et al., 2014; Heathers, 2014; Lehrer and Gevirtz, 2014; Quintana and Heathers, 2014; Quintana et al., 2016a; Laborde et al., 2017). In population with known fast breathing rate, like children, respiratory depth, and frequency are particularly associated with heart rate and its fluctuations and may influence HRV data independently of cardiac autonomic activity (Eckberg, 2009; Quintana et al., 2016b). Also in our study, we observed significant correlation between children’s respiratory rate and HR, nevertheless, the correlation between HRV and HR was stronger than the association between HRV and breathing rate, and the statistical analysis revealed that respiratory rate was redundant in the multiple regression analysis. In our recent study, we have shown that the influence of breathing rate on HRV appears to be, at least in part, HR dependent, i.e., after HRV correction for prevailing HR, the correlation between breathing rate and HRV decreases (Gąsior et al., 2016).

In the present study, we calculated VLF power from short-term ECG recordings (i.e., lasting 5 min) which may be problematic from methodological point of view (Task Force, 1996; Heathers, 2014). However, this parameter was also considered in the previous study concerning reference values for short-term (5 min recordings) standard HRV in children (Michels et al., 2013). Shaffer and Ginsberg in an overview paper concerning HRV metrics and norms state that “the VLF band requires a recording period of at least 5 min” (Shaffer and Ginsberg, 2017). Moreover, in our previous studies addressing large populations of patients after myocardial infarction, VLF parameter calculated from 512 R-R intervals turned out to be the strongest predictor of different causes of death (Sacha et al., 2013a, 2014). Therefore, we included this parameter in the present analysis despite the short-term nature of ECG recordings. This may enable future studies aiming to explore its clinical value in pediatric populations with various disorders and consequently may help to understand the nature of this parameter.

To make our data pragmatic and comparable for other studies: (i) we calculated short-term time- and frequency-domain HRV parameters by using actual version of a very popular and free tool: Kubios HRV Standard ver. 3.0.2 (Tarvainen et al., 2014, 2017); (ii) in spectral analysis, we used frequency bands up to 0,5 Hz which is appropriate for children populations; (iii) we presented frequency-domain HRV parameters calculated with both fast Fourier transform and autoregressive method; and (iv) we provide the Excel sheet calculator to facilitate computation and assessment of corrected HRV parameters.

### Limitations

A limitation of this study is the relatively narrow age range for which these normal limits can be applied, i.e., 6–13 years. However, this age period corresponds to the considerable developmental changes in both circulatory and neurological systems, and therefore the HRV normative values for this life period may be very useful in different clinical scenarios.

Finally, the norms can only be applied in Caucasian children. Caution is needed in applying the normative HRV values for other populations as the significant ethnic differences of HRV in children and youth were shown (Wang et al., 2005; Reed et al., 2006; Eyre et al., 2013). Future studies using approach considering differences in average HR may help to identify ethnic differences in corrected HRV indices.

## Conclusion

This study provides HRV normative values for school-aged children which have been developed independently of their major determinants, especially related to average HR. An Excel sheet calculator is provided to increase accessibility (Supplementary Table S11, Sheet “Corrected HRV Calculator”) and simplifies determination of HRV parameters from an individual child and if values are within normal limits.

## Author Contributions

JG, JS, and MP conceived and designed the experiment. JG and MP acquired the data. AT, TK, and BW analyzed the data for ECG assessment. JZ analyzed the data for respiratory rate calculations. JG and JS analyzed the data for HRV and statistical analysis. JG, JS, MP, JZ, PJ, AT, TK, BW, and MD interpreted the data. JG, JS, MP, JZ, PJ, AT, TK, BW, and MD drafted the work and revised it critically for important intellectual content. JG, JS, MP, JZ, PJ, AT, TK, BW, and MD approved the final version of the manuscript to be published.

## Conflict of Interest Statement

The authors declare that the research was conducted in the absence of any commercial or financial relationships that could be construed as a potential conflict of interest.
