# Supplementary material for: Normative Values for Heart Rate Variability Parameters in School-Aged Children: Simple Approach Considering Differences in Average Heart Rate
Source: Front Physiol. 2018 Oct 24;9:1495. doi: 10.3389/fphys.2018.01495 (PMC6207594; doi:10.3389/fphys.2018.01495)
Supplement: Supplementary file 1 [file Table_1.DOCX]

**Table S1**. Determinants of standard frequency-domain HRV parameters obtained with the autoregressive method.

| Standard HRV parameter | Determinant | Parameters of multiple regression analysis | | | | | |
| --- | --- | --- | --- | --- | --- | --- | --- |
|  |  | β | p | Partial correlation | Multiple R2 | F-test | p |
| VLF (ln) | HR | -0.64 | <0.001 | -0.61 | 0.40 | 67.5 | <0.001 |
|  | Age (ln) | -0.25 | <0.001 | -0.29 |  |  |  |
|  | Sex | 0.07 | 0.13 | 0.09 |  |  |  |
| LF (ln) | HR | -0.62 | <0.001 | -0.59 | 0.37 | 59.9 | <0.001 |
|  | Age (ln) | -0.18 | <0.001 | -0.21 |  |  |  |
|  | Sex | 0.07 | 0.13 | 0.09 |  |  |  |
| HF (ln) | HR | -0.72 | <0.001 | -0.69 | 0.48 | 93.3 | <0.001 |
|  | Age (ln) | -0.24 | <0.001 | -0.30 |  |  |  |
|  | Sex | -0.01 | 0.95 | -0.01 |  |  |  |
| TP_1_ (VLF+LF+HF) (ln) | HR | -0.71 | <0.001 | -0.68 | 0.47 | 92.5 | <0.001 |
|  | Age (ln) | -0.22 | <0.001 | -0.28 |  |  |  |
|  | Sex | 0.02 | 0.57 | 0.03 |  |  |  |
| TP_2_ (LF+HF) (ln) | HR | -0.71 | <0.001 | -0.68 | 0.47 | 92.1 | <0.001 |
|  | Age (ln) | -0.22 | <0.001 | -0.28 |  |  |  |
|  | Sex | 0.02 | 0.62 | 0.03 |  |  |  |
| LF/HF (ln) | HR | 0.33 | <0.001 | 0.32 | 0.11 | 12.0 | <0.001 |
|  | Age (ln) | 0.15 | <0.01 | 0.15 |  |  |  |
|  | Sex | 0.10 | 0.06 | 0.11 |  |  |  |
| nLF | HR | 0.34 | <0.001 | 0.32 | 0.11 | 12.7 | <0.001 |
|  | Age (ln) | 0.16 | <0.01 | 0.16 |  |  |  |
|  | Sex | 0.10 | 0.06 | 0.11 |  |  |  |
| nHF | HR | -0.34 | <0.001 | -0.33 | 0.11 | 12.7 | <0.001 |
|  | Age (ln) | -0.16 | <0.01 | -0.16 |  |  |  |
|  | Sex | -0.10 | 0.06 | -0.11 |  |  |  |
