# Supplementary material for: Normative Values for Heart Rate Variability Parameters in School-Aged Children: Simple Approach Considering Differences in Average Heart Rate
Source: Front Physiol. 2018 Oct 24;9:1495. doi: 10.3389/fphys.2018.01495 (PMC6207594; doi:10.3389/fphys.2018.01495)
Supplement: Supplementary file 2 [file Table_2.DOCX]

The group was divided into four subgroups according to the age: (I) 6 ≤ age < 8 years; (II) 8 ≤ age < 10 years; (III) 10 ≤ age < 12 years and (IV) 12 ≤ age < 14 years. Number of children in the consecutive age subgroups were as follows: 65 (♂/♀: 33/32), 87 (43/44), 89 (42/47) and 71 (35/36). There was no significant difference in gender distribution between these age subgroups (Pearson’s Chi2 = 0.21; p = 0.98). There were no significant differences in standard HRV parameters between the age subgroups (p value > 0.06 for all).

**Table S2**. Determinants of standard time-domain HRV parameters in children aged 6-7 years.

| Standard HRV parameter | Determinant | Parameters of multiple regression analysis | | | | | |
| --- | --- | --- | --- | --- | --- | --- | --- |
|  |  | β | p | Partial correlation | Multiple R2 | F-test | p |
| SDNN (ln) | HR | -0.76 | <0.001 | -0.76 | 0.60 | 29.9 | <0.001 |
|  | Age (ln) | 0.08 | 0.36 | 0.12 |  |  |  |
|  | Sex | -0.06 | 0.46 | -0.10 |  |  |  |
| RMSSD (ln) | HR | -0.84 | <0.001 | -0.83 | 0.70 | 49.2 | <0.001 |
|  | Age (ln) | 0.01 | 0.94 | 0.01 |  |  |  |
|  | Sex | 0.01 | 0.93 | 0.01 |  |  |  |
| pNN50 (ln) | HR | -0.75 | <0.001 | -0.75 | 0.58 | 28.2 | <0.001 |
|  | Age (ln) | 0.06 | 0.49 | 0.09 |  |  |  |
|  | Sex | -0.02 | 0.82 | -0.03 |  |  |  |
