# Supplementary material for: Normative Values for Heart Rate Variability Parameters in School-Aged Children: Simple Approach Considering Differences in Average Heart Rate
Source: Front Physiol. 2018 Oct 24;9:1495. doi: 10.3389/fphys.2018.01495 (PMC6207594; doi:10.3389/fphys.2018.01495)
Supplement: Supplementary file 3 [file Table_3.DOCX]

**Table S3.** Determinants of standard frequency-domain HRV parameters obtained with the fast Fourier transform (FFT) and the autoregressive method (AR) in children aged 6-7 years.

| Standard HRV parameter | Determinant | Parameters of multiple regression analysis | | | | | |
| --- | --- | --- | --- | --- | --- | --- | --- |
|  |  | β | p | Partial correlation | Multiple R2 | F-test | p |
| _FFT_ VLF (ln) | HR | -0.57 | <0.001 | -0.56 | 0.33 | 10.0 | <0.001 |
|  | Age (ln) | 0.06 | 0.61 | 0.07 |  |  |  |
|  | Sex | -0.13 | 0.25 | -0.15 |  |  |  |
| _FFT_ LF (ln) | HR | -0.62 | <0.001 | -0.61 | 0.40 | 13.4 | <0.001 |
|  | Age (ln) | 0.07 | 0.52 | 0.08 |  |  |  |
|  | Sex | -0.07 | 0.49 | -0.09 |  |  |  |
| _FFT_ HF (ln) | HR | -0.69 | <0.001 | -0.68 | 0.50 | 19.9 | <0.001 |
|  | Age (ln) | 0.09 | 0.33 | 0.13 |  |  |  |
|  | Sex | -0.05 | 0.61 | -0.07 |  |  |  |
| _FFT_ TP_1_ (VLF+LF+HF) (ln) | HR | -0.69 | <0.001 | -0.69 | 0.50 | 20.2 | <0.001 |
|  | Age (ln) | 0.09 | 0.35 | 0.12 |  |  |  |
|  | Sex | -0.07 | 0.47 | -0.09 |  |  |  |
| _FFT_ TP_2_ (LF+HF) (ln) | HR | -0.69 | <0.001 | -0.68 | 0.49 | 19.6 | <0.001 |
|  | Age (ln) | 0.09 | 0.35 | 0.12 |  |  |  |
|  | Sex | -0.06 | 0.50 | -0.09 |  |  |  |
| _FFT_ LF/HF (ln) | HR | 0.18 | 0.18 | 0.17 | 0.04 | 0.8 | 0.49 |
|  | Age (ln) | -0.05 | 0.70 | -0.05 |  |  |  |
|  | Sex | -0.03 | 0.82 | -0.03 |  |  |  |
| _FFT_ nLF | HR | 0.19 | 0.14 | 0.19 | 0.05 | 0.9 | 0.40 |
|  | Age (ln) | -0.05 | 0.70 | -0.05 |  |  |  |
|  | Sex | -0.04 | 0.77 | -0.04 |  |  |  |
| _FFT_ nHF | HR | -0.19 | 0.15 | -0.19 | 0.05 | 0.9 | 0.41 |
|  | Age (ln) | 0.05 | 0.69 | 0.05 |  |  |  |
|  | Sex | 0.04 | 0.79 | 0.03 |  |  |  |
| _AR_ VLF (ln) | HR | -0.71 | <0.001 | -0.70 | 0.51 | 20.8 | <0.001 |
|  | Age (ln) | 0.01 | 0.97 | 0.01 |  |  |  |
|  | Sex | -0.01 | 0.93 | -0.01 |  |  |  |
| _AR_ LF (ln) | HR | -0.69 | <0.001 | -0.68 | 0.49 | 19.8 | <0.001 |
|  | Age (ln) | 0.09 | 0.37 | 0.12 |  |  |  |
|  | Sex | -0.08 | 0.39 | -0.11 |  |  |  |
| _AR_ HF (ln) | HR | -0.72 | <0.001 | -0.71 | 0.54 | 23.5 | <0.001 |
|  | Age (ln) | 0.09 | 0.30 | 0.13 |  |  |  |
|  | Sex | -0.05 | 0.57 | -0.07 |  |  |  |
| _AR_ TP_1_ (VLF+LF+HF) (ln) | HR | -0.73 | <0.001 | -0.73 | 0.55 | 25.0 | <0.001 |
|  | Age (ln) | 0.09 | 0.30 | 0.13 |  |  |  |
|  | Sex | -0.07 | 0.45 | -0.10 |  |  |  |
| _AR_ TP_2_ (LF+HF) (ln) | HR | -0.73 | <0.001 | -0.72 | 0.55 | 24.6 | <0.001 |
|  | Age (ln) | 0.10 | 0.28 | 0.14 |  |  |  |
|  | Sex | -0.07 | 0.44 | -0.10 |  |  |  |
| _AR_ LF/HF (ln) | HR | 0.18 | 0.19 | 0.17 | 0.04 | 0.8 | 0.51 |
|  | Age (ln) | -0.03 | 0.82 | -0.03 |  |  |  |
|  | Sex | -0.04 | 0.75 | -0.04 |  |  |  |
| _AR_ nLF | HR | 0.21 | 0.11 | 0.21 | 0.06 | 1.2 | 0.33 |
|  | Age (ln) | -0.03 | 0.82 | -0.03 |  |  |  |
|  | Sex | -0.06 | 0.66 | -0.06 |  |  |  |
| _AR_ nHF | HR | -0.21 | 0.11 | -0.21 | 0.06 | 1.2 | 0.33 |
|  | Age (ln) | 0.03 | 0.81 | 0.03 |  |  |  |
|  | Sex | 0.06 | 0.67 | 0.05 |  |  |  |
