# Supplementary material for: Normative Values for Heart Rate Variability Parameters in School-Aged Children: Simple Approach Considering Differences in Average Heart Rate
Source: Front Physiol. 2018 Oct 24;9:1495. doi: 10.3389/fphys.2018.01495 (PMC6207594; doi:10.3389/fphys.2018.01495)
Supplement: Supplementary file 4 [file Table_4.DOCX]

**Table S4**. Determinants of standard time-domain HRV parameters in children aged 8-9 years.

| Standard HRV parameter | Determinant | Parameters of multiple regression analysis | | | | | |
| --- | --- | --- | --- | --- | --- | --- | --- |
|  |  | β | p | Partial correlation | Multiple R2 | F-test | p |
| SDNN (ln) | HR | -0.59 | <0.001 | -0.60 | 0.40 | 18.6 | <0.001 |
|  | Age (ln) | -0.01 | 0.98 | -0.01 |  |  |  |
|  | Sex | 0.13 | 0.13 | 0.17 |  |  |  |
| RMSSD (ln) | HR | -0.70 | <0.001 | -0.70 | 0.51 | 29.3 | <0.001 |
|  | Age (ln) | -0.02 | 0.75 | -0.04 |  |  |  |
|  | Sex | 0.06 | 0.43 | 0.09 |  |  |  |
| pNN50 (ln) | HR | -0.72 | <0.001 | -0.73 | 0.56 | 34.2 | <0.001 |
|  | Age (ln) | -0.17 | <0.05 | -0.25 |  |  |  |
|  | Sex | 0.01 | 0.87 | 0.02 |  |  |  |
