# Supplementary material for: Normative Values for Heart Rate Variability Parameters in School-Aged Children: Simple Approach Considering Differences in Average Heart Rate
Source: Front Physiol. 2018 Oct 24;9:1495. doi: 10.3389/fphys.2018.01495 (PMC6207594; doi:10.3389/fphys.2018.01495)
Supplement: Supplementary file 5 [file Table_5.DOCX]

**Table S5**. Determinants of standard frequency-domain HRV parameters obtained with the fast Fourier transform (FFT) and the autoregressive method (AR) in children aged 8-9 years.

| Standard HRV parameter | Determinant | Parameters of multiple regression analysis | | | | | |
| --- | --- | --- | --- | --- | --- | --- | --- |
|  |  | β | p | Partial correlation | Multiple R2 | F-test | p |
| _FFT_ VLF (ln) | HR | -0.32 | <0.01 | -0.33 | 0.19 | 6.3 | <0.001 |
|  | Age (ln) | -0.15 | 0.15 | -0.16 |  |  |  |
|  | Sex | 0.21 | 0.06 | 0.21 |  |  |  |
| _FFT_ LF (ln) | HR | -0.45 | <0.001 | -0.48 | 0.35 | 14.6 | <0.001 |
|  | Age (ln) | -0.01 | 0.88 | -0.02 |  |  |  |
|  | Sex | 0.30 | <0.01 | 0.34 |  |  |  |
| _FFT_ HF (ln) | HR | -0.57 | <0.001 | -0.57 | 0.36 | 15.2 | <0.001 |
|  | Age (ln) | 0.04 | 0.64 | 0.05 |  |  |  |
|  | Sex | 0.08 | 0.37 | 0.10 |  |  |  |
| _FFT_ TP_1_ (VLF+LF+HF) (ln) | HR | -0.55 | <0.001 | -0.56 | 0.37 | 16.4 | <0.001 |
|  | Age (ln) | 0.03 | 0.77 | 0.03 |  |  |  |
|  | Sex | 0.17 | 0.07 | 0.20 |  |  |  |
| _FFT_ TP_2_ (LF+HF) (ln) | HR | -0.56 | <0.001 | -0.57 | 0.38 | 16.7 | <0.001 |
|  | Age (ln) | 0.03 | 0.73 | 0.04 |  |  |  |
|  | Sex | 0.16 | 0.08 | 0.19 |  |  |  |
| _FFT_ LF/HF (ln) | HR | 0.33 | <0.01 | 0.33 | 0.15 | 5.0 | <0.01 |
|  | Age (ln) | -0.09 | 0.38 | -0.10 |  |  |  |
|  | Sex | 0.28 | <0.01 | 0.29 |  |  |  |
| _FFT_ nLF | HR | 0.32 | <0.01 | 0.33 | 0.16 | 5.1 | <0.01 |
|  | Age (ln) | -0.08 | 0.46 | -0.08 |  |  |  |
|  | Sex | 0.29 | <0.01 | 0.30 |  |  |  |
| _FFT_ nHF | HR | -0.32 | <0.01 | -0.33 | 0.16 | 5.1 | <0.01 |
|  | Age (ln) | 0.08 | 0.46 | 0.08 |  |  |  |
|  | Sex | -0.30 | <0.01 | -0.30 |  |  |  |
| _AR_ VLF (ln) | HR | -0.47 | <0.001 | -0.49 | 0.33 | 13.7 | <0.001 |
|  | Age (ln) | -0.07 | 0.45 | -0.08 |  |  |  |
|  | Sex | 0.25 | <0.01 | 0.29 |  |  |  |
| _AR_ LF (ln) | HR | -0.42 | <0.001 | -0.44 | 0.31 | 12.3 | <0.001 |
|  | Age (ln) | -0.06 | 0.55 | -0.07 |  |  |  |
|  | Sex | 0.29 | <0.01 | 0.32 |  |  |  |
| _AR_ HF (ln) | HR | -0.61 | <0.001 | -0.60 | 0.38 | 17.2 | <0.001 |
|  | Age (ln) | 0.03 | 0.75 | 0.04 |  |  |  |
|  | Sex | 0.05 | 0.57 | 0.06 |  |  |  |
| _AR_ TP_1_ (VLF+LF+HF) (ln) | HR | -0.58 | <0.001 | -0.58 | 0.38 | 17.2 | <0.001 |
|  | Age (ln) | 0.01 | 0.96 | 0.01 |  |  |  |
|  | Sex | 0.14 | 0.13 | 0.17 |  |  |  |
| _AR_ TP_2_ (LF+HF) (ln) | HR | -0.58 | <0.001 | -0.58 | 0.38 | 17.1 | <0.001 |
|  | Age (ln) | 0.01 | 0.94 | 0.01 |  |  |  |
|  | Sex | 0.13 | 0.14 | 0.16 |  |  |  |
| _AR_ LF/HF (ln) | HR | 0.40 | <0.001 | 0.41 | 0.22 | 7.9 | <0.001 |
|  | Age (ln) | -0.12 | 0.21 | -0.14 |  |  |  |
|  | Sex | 0.33 | <0.01 | 0.34 |  |  |  |
| _AR_ nLF | HR | 0.39 | <0.001 | 0.40 | 0.22 | 7.7 | <0.001 |
|  | Age (ln) | -0.11 | 0.28 | -0.12 |  |  |  |
|  | Sex | 0.34 | <0.01 | 0.35 |  |  |  |
| _AR_ nHF | HR | -0.39 | <0.001 | -0.40 | 0.22 | 7.7 | <0.001 |
|  | Age (ln) | 0.11 | 0.28 | 0.12 |  |  |  |
|  | Sex | -0.34 | <0.01 | -0.35 |  |  |  |
