# Supplementary material for: Normative Values for Heart Rate Variability Parameters in School-Aged Children: Simple Approach Considering Differences in Average Heart Rate
Source: Front Physiol. 2018 Oct 24;9:1495. doi: 10.3389/fphys.2018.01495 (PMC6207594; doi:10.3389/fphys.2018.01495)
Supplement: Supplementary file 6 [file Table_6.DOCX]

**Table S6**. Determinants of standard time-domain HRV parameters in children aged 10-11 years.

| Standard HRV parameter | Determinant | Parameters of multiple regression analysis | | | | | |
| --- | --- | --- | --- | --- | --- | --- | --- |
|  |  | β | p | Partial correlation | Multiple R2 | F-test | p |
| SDNN (ln) | HR | -0.74 | <0.001 | -0.74 | 0.58 | 39.2 | <0.001 |
|  | Age (ln) | -0.07 | 0.32 | -0.11 |  |  |  |
|  | Sex | 0.05 | 0.46 | 0.08 |  |  |  |
| RMSSD (ln) | HR | -0.79 | <0.001 | -0.78 | 0.63 | 48.8 | <0.001 |
|  | Age (ln) | <-0.01 | 0.99 | <-0.01 |  |  |  |
|  | Sex | 0.03 | 0.63 | 0.05 |  |  |  |
| pNN50 (ln) | HR | -0.71 | <0.001 | -0.71 | 0.52 | 30.8 | <0.001 |
|  | Age (ln) | -0.01 | 0.87 | -0.02 |  |  |  |
|  | Sex | 0.04 | 0.57 | 0.06 |  |  |  |
