# Supplementary material for: Normative Values for Heart Rate Variability Parameters in School-Aged Children: Simple Approach Considering Differences in Average Heart Rate
Source: Front Physiol. 2018 Oct 24;9:1495. doi: 10.3389/fphys.2018.01495 (PMC6207594; doi:10.3389/fphys.2018.01495)
Supplement: Supplementary file 7 [file Table_7.DOCX]

**Table S7**. Determinants of standard frequency-domain HRV parameters obtained with the fast Fourier transform (FFT) and the autoregressive method (AR) in children aged 10-11 years.

| Standard HRV parameter | Determinant | Parameters of multiple regression analysis | | | | | |
| --- | --- | --- | --- | --- | --- | --- | --- |
|  |  | β | p | Partial correlation | Multiple R2 | F-test | p |
| _FFT_ VLF (ln) | HR | -0.59 | <0.001 | -0.59 | 0.40 | 18.8 | <0.001 |
|  | Age (ln) | -0.12 | 0.16 | -0.15 |  |  |  |
|  | Sex | 0.07 | 0.45 | 0.08 |  |  |  |
| _FFT_ LF (ln) | HR | -0.60 | <0.001 | -0.62 | 0.46 | 24.3 | <0.001 |
|  | Age (ln) | -0.21 | <0.01 | -0.28 |  |  |  |
|  | Sex | 0.08 | 0.34 | 0.10 |  |  |  |
| _FFT_ HF (ln) | HR | -0.75 | <0.001 | -0.75 | 0.58 | 39.6 | <0.001 |
|  | Age (ln) | -0.01 | 0.85 | -0.02 |  |  |  |
|  | Sex | 0.04 | 0.63 | 0.05 |  |  |  |
| _FFT_ TP_1_ (VLF+LF+HF) (ln) | HR | -0.73 | <0.001 | -0.74 | 0.58 | 38.4 | <0.001 |
|  | Age (ln) | -0.09 | 0.21 | -0.14 |  |  |  |
|  | Sex | 0.05 | 0.48 | 0.08 |  |  |  |
| _FFT_ TP_2_ (LF+HF) (ln) | HR | -0.73 | <0.001 | -0.74 | 0.58 | 38.4 | <0.001 |
|  | Age (ln) | -0.09 | 0.21 | -0.14 |  |  |  |
|  | Sex | 0.05 | 0.48 | 0.08 |  |  |  |
| _FFT_ LF/HF (ln) | HR | 0.36 | <0.001 | 0.36 | 0.18 | 6.2 | <0.001 |
|  | Age (ln) | -0.29 | <0.01 | -0.30 |  |  |  |
|  | Sex | 0.06 | 0.57 | 0.06 |  |  |  |
| _FFT_ nLF | HR | 0.36 | <0.001 | 0.36 | 0.18 | 6.3 | <0.001 |
|  | Age (ln) | -0.29 | <0.01 | -0.30 |  |  |  |
|  | Sex | 0.05 | 0.63 | 0.05 |  |  |  |
| _FFT_ nHF | HR | -0.36 | <0.001 | -0.36 | 0.18 | 6.3 | <0.001 |
|  | Age (ln) | 0.28 | <0.01 | 0.30 |  |  |  |
|  | Sex | -0.05 | 0.60 | -0.06 |  |  |  |
| _AR_ VLF (ln) | HR | -0.66 | <0.001 | -0.67 | 0.49 | 27.7 | <0.001 |
|  | Age (ln) | -0.13 | 0.09 | -0.18 |  |  |  |
|  | Sex | 0.04 | 0.58 | 0.06 |  |  |  |
| _AR_ LF (ln) | HR | -0.61 | <0.001 | -0.62 | 0.44 | 21.8 | <0.001 |
|  | Age (ln) | -0.16 | 0.05 | -0.21 |  |  |  |
|  | Sex | 0.06 | 0.47 | 0.08 |  |  |  |
| _AR_ HF (ln) | HR | -0.75 | <0.001 | -0.74 | 0.58 | 38.7 | <0.001 |
|  | Age (ln) | -0.03 | 0.68 | -0.05 |  |  |  |
|  | Sex | 0.04 | 0.61 | 0.06 |  |  |  |
| _AR_ TP_1_ (VLF+LF+HF) (ln) | HR | -0.73 | <0.001 | -0.73 | 0.56 | 36.4 | <0.001 |
|  | Age (ln) | -0.08 | 0.27 | -0.12 |  |  |  |
|  | Sex | 0.05 | 0.53 | 0.07 |  |  |  |
| _AR_ TP_2_ (LF+HF) (ln) | HR | -0.72 | <0.001 | -0.73 | 0.56 | 36.0 | <0.001 |
|  | Age (ln) | -0.08 | 0.29 | -0.12 |  |  |  |
|  | Sex | 0.05 | 0.54 | 0.07 |  |  |  |
| _AR_ LF/HF (ln) | HR | 0.35 | <0.001 | 0.35 | 0.14 | 4.7 | <0.01 |
|  | Age (ln) | -0.19 | 0.06 | -0.20 |  |  |  |
|  | Sex | 0.03 | 0.79 | 0.03 |  |  |  |
| _AR_ nLF | HR | 0.36 | <0.001 | 0.35 | 0.14 | 4.7 | <0.01 |
|  | Age (ln) | -0.19 | 0.07 | -0.20 |  |  |  |
|  | Sex | 0.02 | 0.84 | 0.02 |  |  |  |
| _AR_ nHF | HR | -0.36 | <0.001 | -0.35 | 0.14 | 4.7 | <0.01 |
|  | Age (ln) | 0.19 | 0.07 | 0.20 |  |  |  |
|  | Sex | -0.02 | 0.84 | -0.02 |  |  |  |
