# Supplementary material for: Normative Values for Heart Rate Variability Parameters in School-Aged Children: Simple Approach Considering Differences in Average Heart Rate
Source: Front Physiol. 2018 Oct 24;9:1495. doi: 10.3389/fphys.2018.01495 (PMC6207594; doi:10.3389/fphys.2018.01495)
Supplement: Supplementary file 8 [file Table_8.DOCX]

**Table S8**. Determinants of standard time-domain HRV parameters in children aged 12-13 years.

| Standard HRV parameter | Determinant | Parameters of multiple regression analysis | | | | | |
| --- | --- | --- | --- | --- | --- | --- | --- |
|  |  | β | p | Partial correlation | Multiple R2 | F-test | p |
| SDNN (ln) | HR | -0.70 | <0.001 | -0.69 | 0.48 | 20.8 | <0.001 |
|  | Age (ln) | -0.05 | 0.62 | -0.06 |  |  |  |
|  | Sex | -0.04 | 0.65 | -0.06 |  |  |  |
| RMSSD (ln) | HR | -0.77 | <0.001 | -0.76 | 0.58 | 31.2 | <0.001 |
|  | Age (ln) | -0.02 | 0.84 | -0.02 |  |  |  |
|  | Sex | -0.06 | 0.50 | -0.08 |  |  |  |
| pNN50 (ln) | HR | -0.68 | <0.001 | -0.68 | 0.51 | 22.6 | <0.001 |
|  | Age (ln) | 0.11 | 0.25 | 0.14 |  |  |  |
|  | Sex | -0.11 | 0.22 | -0.15 |  |  |  |
