# Supplementary material for: Normative Values for Heart Rate Variability Parameters in School-Aged Children: Simple Approach Considering Differences in Average Heart Rate
Source: Front Physiol. 2018 Oct 24;9:1495. doi: 10.3389/fphys.2018.01495 (PMC6207594; doi:10.3389/fphys.2018.01495)
Supplement: Supplementary file 9 [file Table_9.DOCX]

**Table S9**. Determinants of standard frequency-domain HRV parameters obtained with the fast Fourier transform (FFT) and the autoregressive method (AR) in children aged 12-13 years.

| Standard HRV parameter | Determinant | Parameters of multiple regression analysis | | | | | |
| --- | --- | --- | --- | --- | --- | --- | --- |
|  |  | β | p | Partial correlation | Multiple R2 | F-test | p |
| _FFT_ VLF (ln) | HR | -0.38 | <0.01 | -0.38 | 0.14 | 3.7 | <0.05 |
|  | Age (ln) | -0.05 | 0.69 | -0.05 |  |  |  |
|  | Sex | 0.02 | 0.84 | 0.03 |  |  |  |
| _FFT_ LF (ln) | HR | -0.58 | <0.001 | -0.57 | 0.33 | 10.9 | <0.001 |
|  | Age (ln) | -0.08 | 0.46 | -0.09 |  |  |  |
|  | Sex | -0.02 | 0.88 | -0.02 |  |  |  |
| _FFT_ HF (ln) | HR | -0.68 | <0.001 | -0.67 | 0.45 | 18.3 | <0.001 |
|  | Age (ln) | -0.04 | 0.69 | -0.05 |  |  |  |
|  | Sex | -0.06 | 0.55 | -0.07 |  |  |  |
| _FFT_ TP_1_ (VLF+LF+HF) (ln) | HR | -0.67 | <0.001 | -0.66 | 0.44 | 17.4 | <0.001 |
|  | Age (ln) | -0.07 | 0.45 | -0.09 |  |  |  |
|  | Sex | -0.04 | 0.71 | -0.05 |  |  |  |
| _FFT_ TP_2_ (LF+HF) (ln) | HR | -0.68 | <0.001 | -0.67 | 0.45 | 17.9 | <0.001 |
|  | Age (ln) | -0.07 | 0.44 | -0.09 |  |  |  |
|  | Sex | -0.04 | 0.67 | -0.05 |  |  |  |
| _FFT_ LF/HF (ln) | HR | 0.40 | <0.001 | 0.39 | 0.16 | 4.3 | <0.01 |
|  | Age (ln) | -0.03 | 0.79 | -0.03 |  |  |  |
|  | Sex | 0.07 | 0.54 | 0.07 |  |  |  |
| _FFT_ nLF | HR | 0.39 | <0.001 | 0.39 | 0.17 | 4.4 | <0.01 |
|  | Age (ln) | -0.04 | 0.68 | -0.05 |  |  |  |
|  | Sex | 0.08 | 0.49 | 0.09 |  |  |  |
| _FFT_ nHF | HR | -0.39 | <0.001 | -0.39 | 0.17 | 4.5 | <0.01 |
|  | Age (ln) | 0.05 | 0.68 | 0.05 |  |  |  |
|  | Sex | -0.08 | 0.49 | -0.08 |  |  |  |
| _AR_ VLF (ln) | HR | -0.58 | <0.001 | -0.57 | 0.33 | 11.0 | <0.001 |
|  | Age (ln) | -0.12 | 0.24 | -0.14 |  |  |  |
|  | Sex | -0.04 | 0.68 | -0.05 |  |  |  |
| _AR_ LF (ln) | HR | -0.60 | <0.001 | -0.59 | 0.36 | 12.4 | <0.001 |
|  | Age (ln) | -0.02 | 0.86 | -0.02 |  |  |  |
|  | Sex | -0.01 | 0.90 | -0.02 |  |  |  |
| _AR_ HF (ln) | HR | -0.68 | <0.001 | -0.67 | 0.46 | 19.1 | <0.001 |
|  | Age (ln) | -0.01 | 0.88 | -0.02 |  |  |  |
|  | Sex | -0.05 | 0.55 | -0.07 |  |  |  |
| _AR_ TP_1_ (VLF+LF+HF) (ln) | HR | -0.69 | <0.001 | -0.68 | 0.46 | 19.2 | <0.001 |
|  | Age (ln) | -0.04 | 0.68 | -0.05 |  |  |  |
|  | Sex | -0.04 | 0.70 | -0.05 |  |  |  |
| _AR_ TP_2_ (LF+HF) (ln) | HR | -0.69 | <0.001 | -0.68 | 0.47 | 19.4 | <0.001 |
|  | Age (ln) | -0.03 | 0.72 | -0.04 |  |  |  |
|  | Sex | -0.04 | 0.68 | -0.05 |  |  |  |
| _AR_ LF/HF (ln) | HR | 0.39 | <0.01 | 0.39 | 0.16 | 4.1 | <0.01 |
|  | Age (ln) | <0.01 | 0.99 | <0.01 |  |  |  |
|  | Sex | 0.07 | 0.51 | 0.08 |  |  |  |
| _AR_ nLF | HR | 0.39 | <0.001 | 0.39 | 0.16 | 4.3 | <0.01 |
|  | Age (ln) | -0.02 | 0.90 | -0.02 |  |  |  |
|  | Sex | 0.09 | 0.45 | 0.09 |  |  |  |
| _AR_ nHF | HR | -0.39 | <0.001 | -0.39 | 0.16 | 4.3 | <0.01 |
|  | Age (ln) | 0.02 | 0.89 | 0.02 |  |  |  |
|  | Sex | -0.09 | 0.45 | -0.09 |  |  |  |
