# Supplementary material for: Normative Values for Heart Rate Variability Parameters in School-Aged Children: Simple Approach Considering Differences in Average Heart Rate
Source: Front Physiol. 2018 Oct 24;9:1495. doi: 10.3389/fphys.2018.01495 (PMC6207594; doi:10.3389/fphys.2018.01495)
Supplement: Supplementary file 10 [file Table_10.DOCX]

**Table S10**. Determinants and Cohen’s f^2^ indexes for corrected frequency-domain HRV parameters obtained with the autoregressive method.

| Corrected HRV parameter | Det. | Parameters of multiple regression analysis | | | | | | Cohen’s f^2^ | |
| --- | --- | --- | --- | --- | --- | --- | --- | --- | --- |
|  |  | β | p | PC | M. R2 | F-test | p | Loc. | Comb. |
| corr-VLF | Age (ln) | -0.29 | <0.001 | -0.29 | 0.09 | 14.9 | <0.001 | 0.091 | 0.089 |
|  | Sex | 0.07 | 0.18 | 0.08 |  |  |  | 0.006 |  |
| corr-LF | Age (ln) | -0.17 | <0.01 | -0.18 | 0.04 | 6.7 | <0.01 | 0.031 | 0.031 |
|  | Sex | 0.11 | 0.06 | 0.11 |  |  |  | 0.012 |  |
| corr-HF | Age (ln) | -0.20 | <0.001 | -0.20 | 0.04 | 6.2 | <0.01 | 0.040 | 0.039 |
|  | Sex | 0.02 | 0.69 | 0.02 |  |  |  | 0.001 |  |
| corr-TP_1_  (VLF+LF+HF) | Age (ln) | -0.23 | <0.001 | -0.23 | 0.06 | 9.0 | <0.001 | 0.056 | 0.056 |
|  | Sex | 0.05 | 0.42 | 0.05 |  |  |  | 0.002 |  |
| corr-TP_2_ (LF+HF) | Age (ln) | -0.22 | <0.001 | -0.22 | 0.05 | 8.4 | <0.001 | 0.053 | 0.053 |
|  | Sex | 0.04 | 0.42 | 0.05 |  |  |  | 0.002 |  |
| corr-LF/HF | Age (ln) | 0.13 | <0.05 | 0.13 | 0.02 | 3.5 | <0.05 | 0.018 | 0.017 |
|  | Sex | 0.07 | 0.22 | 0.07 |  |  |  | 0.005 |  |
| corr-nLF | Age (ln) | 0.15 | <0.01 | 0.15 | 0.03 | 5.5 | <0.01 | 0.022 | 0.023 |
|  | Sex | 0.11 | <0.05 | 0.11 |  |  |  | 0.012 |  |
| corr-nHF | Age (ln) | -0.15 | <0.01 | -0.15 | 0.03 | 5.4 | <0.01 | 0.024 | 0.024 |
|  | Sex | -0.10 | 0.06 | -0.10 |  |  |  | 0.009 |  |

Abbreviations: Det. – Determinant; PC – Partial Correlation; M. R2 – Multiple R2; Loc. – local; Comb. – combined. Corrected HRV parameters were calculated as follows: corr-SDNN=SDNN/mRR^2.2, corr-RMSSD=RMSSD/mRR^3.0, corr-pNN50=pNN50/mRR^5.0, FFT corr-VLF=VLF/mRR^3.0, AR corr-VLF=VLF/mRR^4.0, for both FFT and AR: corr-LF=LF/mRR^4.0, corr-HF=HF/mRR^5.0, corr-TP_1_=TP_1_/mRR^5.0, corr-TP_2_=TP_2_/mRR^5.0, corr-LF/HF=LF/HF*mRR^1.0, corr-nLF=nLF*mRR^1.0 and corr-nHF=nHF/mRR^0.5.
